# Evaluating the Extent of Pregravid Risk Factors of Gestational Diabetes Mellitus in Women in Tehran

**Published:** 2011-06-01

**Authors:** S Sh Hoseini, S Hantoushzadeh, S Shoar

**Affiliations:** 1 Department of Perinatalogy, Vali-e-Asr Reproductive Health Research Centre, Imam Khomeini Hospital, Tehran University of Medical Sciences, Tehran, Iran

**Keywords:** Pregravid, Risk factors, Gestational diabetes mellitus, Iran

## Abstract

**Background:**

Gestational diabetes mellitus (GDM) is associated with maternal and fetal complications. Specific outcomes in previous pregnancies are considered as risk factors for GDM in the consecutive pregnancies. The aim of this study was to evaluate the pregravid risk factors of GDM in multigravid women.

**Methods:**

We conducted a retrospective cross sectional study on 114 multigravid women with GDM without previous history of diabetes and compared them with non-diabetic controls. We used modified criteria of Carpenter and Coustan for screening. Risk factors were obtained from medical records of individuals.

**Results:**

We found that women of 26 years and older who had previous neonates with birth weight more than 3800 gram and those affected with hypothyroidism or chronic hypertension were at risk for GDM. The difference of the number of preterm birth in GDM women and healthy controls was statistically significant (p=0.05). There was no significant difference between the numbers of pregnancies, parity, previous fetal and neonatal death and abortion number between patients and the control group.

**Conclusion:**

Our results show that maternal age over 26 years, birth weight of previous neonate more than 3800 g, hypothyroidism, chronic hypertension and probably history of preterm birth are significant risk factors for GDM.

## Introduction

Gestational diabetes mellitus (GDM) is defined as diabetes which is first discovered or has its onset during pregnancy. The prevalence of this disease varies between populations from 1% to 16% because of the differences in the populations and the diagnostic methods used.[1][2][3] In Iran, according to separate studies by Hossein-nezhad and Khooshideh, GDM prevalence was estimated 4-8.9%.[4][5][6] GDM is not only associated with maternal complications but also with fetal problems that could impose a great burden on health systems. Maternal complications include pregnancy induced hypertension and preeclampsia, premature ruptures of membranes, preterm birth and the increased risk of cesarean section. Affected women are in an increased risk for diabetes in the following years.[7][8][9] The fetuses whose mothers have experienced gestational diabetes mellitus are at increased risk of macrosomia and being large for gestational age which put them in the risk of birth trauma, shoulder distocia, neonatal hypoglycemia and neonatal death.[10]

In medical sciences, identification of risk factors is very important. If we can determine the risk factors, we can identify the population at risk and design some interventions to prevent the disease. Moreover, if the disease established, we can diagnose it in early stages and prevent its progression and decrease its complications. There are lots of studies which have evaluated the common risk factors of GDM. The obesity before pregnancy and high weight gain during pregnancy, family history of diabetes mellitus, high maternal age, some ethnic backgrounds and previous diagnosis of gestational diabetes, impaired fasting glucose or impaired glucose tolerance predispose women to GDM.[4][11][12][13] In this study, we evaluated the previous history of those multiparous women with no previous history of overt or gestational diabetes mellitus and compared them with the healthy multiparous control group to find potential risk factors which are associated with GDM.

## Materials and Methods

We conducted a retrospective cross sectional study using the medical records of pregnant women came to Antenatal Clinic of Imam Khomeini Hospital, the largest teaching hospital of Tehran University of Medical Sciences in Tehran, Iran, over a period of two years. The patient’s files were randomly selected. Primigravid women and those with previous history of overt diabetes or GDM were excluded from the study. The test group was consisted of 114 multiparous pregnant women with first diagnosis of GDM and the control group was consisted of 113 pregnant healthy ones. We used modified criteria of Carpenter and Coustan for screening. Women without previously diagnosed diabetes underwent fifty gram nonfasting one hour glucose challenge test between 24 to 28 weeks of gestation. Women who had a blood sugar more than 140 mg/dl underwent a 100-gram three hour glucose tolerance test after three days of a high carbohydrate diet. If two or more of the plasma glucose measurements meet or exceed the following thresholds, the diagnosis of GDM was confirmed: fasting plasma glucose=95 mg/dl, one hour plasma glucose=180 mg/dl, two hour plasma glucose=155 mg/dl and three hour plasma glucose=140 mg/dl.[3] Diagnosis of hypothyroidism was made by evaluating the TSH and free T4 levels which were measured at the central laboratory of the hospital. Reference range for TSH was 0.35–4 mU/I for this laboratory. Women with TSH more than 4 and free T4 below the lower limit were considered to have hypothyroidism. Statistical analysis was performed with SPSS software (version 15.0 for windows, Chicago, IL, USA). Means, standard deviations (SDs) and percentages were used to describe continuous and categorical data, respectively. The student’s t-test, for normally distributed data, and Mann-Whitney test, for nonnormally distributed ones, were used to analyze the differences of continuous variables between two groups while the Chi-Square test was used for categorical data. The 2-tailed p-value less than 0.05 was considered to be statistically significant. The study was approved by the Ethics Committee of Imam Khomeini Hospital.

## Results

This study included 114 multiparous women without previous history of GDM or overt diabetes mellitus with first diagnosis of GDM in the current pregnancy and 113 control subjects without any history of diabetes. The mean age of the test group was 31.39±5.56 years (mean±SD) while the mean age of control group was 29.2±5.39 years. We found that risk for GDM increased significantly with increase in maternal age and in women of 26 years and older who had a 2-fold increase in risk for GDM in comparison with those who were younger (OR=2.1, p=0.028, Table 1).

**Table 1:** Comparative analysis of gestational diabetes mellitus risk factors in women in Tehran

	**Number**				**P value**	**Odds ratio**
	**GDM**	**%**	**Control**	**%**		
Maternal age						
<26	16	14	29	26	0.028	2.1
≥26	98	86	84	74			
Fetal death	14	12.3	9	8	0.25	1.6
Neonatal death	4	3.5	7	6.2	0.20	0.55
Hypothyroidism	10	9.3	2	1.8	0.018	5.3
Chronic hypertension	16	14	4	3.5	0.002	4.4

Previous neonate birth weight						
<3800	74	65	107	94	0.001	9.6
≥3800	40	35	6	6			
Abortion Number						
0	71	62.3	74	65.5	0.66 a	
1	30	26.3	26	23		
2	8	7	9	8		
≥3	5	4.4	4	3.5		
Preterm birth number						
0	90	79	99	87.7	0.055 a	
1	19	17	11	9.7		
2	5	4	1	0.9		
≥3	0	0	2	1.8		
Number of previous pregnancies						
1-2	50	43.8	62	54.8	0.10 a	
3-4	48	42.2	39	34.6		
≥5	16	14	12	10.6		
Parity						
0	11	9.6	12	10.6	0.083 a	
1	57	50	70	62		
2	28	24.6	19	16.8		
≥3	18	15.8	12	10.6		

^a^ Mann Whitney test

We assessed the relationship between the birth weight of previous neonate and GDM. The difference between weight of previous neonate in the test (3674±814 g) and control (3080±568 g) groups was statistically significant (p<0.001). Women whose previous neonates weighted more than 3800 g were at higher risk of GDM (OR=9.6, p<0.001, Table 1). The difference between number of pregnancies as well as parity in GDM (3.07±1.22 and 1.5±0.96 respectively) and control subjects (2.88±1.38 and 1.36±1.18 respectively) did not reach the significance threshold (p=0.10 and p=0.083 respectively, Table 1).

The prevalence of previous fetal and neonatal deaths compared between GDM patients (12.3% and 3.5% respectively) and healthy controls (8% and 6.2% respectively) were not statistically significant (p=0.25 and 0.2 respectively, Table 1). We did not find any significant association between the number of previous abortions and GDM in the test (0.57±0.96) and control groups (0.53±0.94) in our study (p=0.66, Table 1). We also assessed the number of preterm birth in GDM women (0.25±0.53) and healthy controls (0.17±0.49) while the difference between the groups was of borderline statistical significance (p=0.055, Table 1).

Furthermore, we evaluated the association between hypothyroidism as well as chronic hypertension with GDM. Women with history of hypothyroidism or chronic hypertension were more prone to get GDM (OR=5.3, p=0.018 and OR=4.4, p=0.002 respectively, Table 1).

## Discussion

Considering that GDM can complicate the maternal and fetal outcomes of pregnancy and can progress to overt diabetes, we tried to assess risk factors which can be used to predict the occurrence of this disease. Also a lot of studies have assessed common risk factors of GDM patients and their pregnancy outcomes, to our knowledge, there are few studies focusing on the previous obstetric and medical history of GDM patients. In this study, we evaluated the previous obstetric and medical history of multiparous women without a history of overt diabetes or GDM.

Our results showed that the risk of GDM rises as the maternal age increases. Women of 26 years and older had a 2-fold increase in risk for GDM (OR=2.1, p=0.028). Different studies have reported different age threshold (25-40 years old) for increased risk of GDM.[5][6][14][15][16][17][18][19][20]

Previous studies have reported that macrosomia, birth weight equal or more than 4000 or 4500 gram in previous pregnancies, is a risk factor for GDM.[5][6][15]

Here we assessed the relation between the birth weight of previous neonate with the risk of GDM and found that women having a previous neonate with birth weight greater than 3800 gram were at increased risk for GDM (OR=9.6, p<0.001). Our results in Iranian women in the present study showed that not only having a history of fetal macrosomia increases the risk of GDM, but also those women who had a neonate with birth weight equal or more than 3800 gram were at risk for this disease. There is evidence showing that significant racial and ethnic variations exist among women with GDM for access to and use of health care, presence of risk factors, and perceptions of health.[21] So the aforementioned results may be due to genetic and racial differences between our study population and those conducted in other geographic areas. Comparison with other studies in different countries is needed to evaluate this new threshold setting.

In our study, the number of pregnancies as well as parity did not have any significant association with GDM, which is consistent with other studies.[5][22] Roman et al. assessed the obstetric and neonatal outcomes in grand multiparity. Although they found that grand multipara had a higher rate of insulindependent gestational diabetes, after doing conditional logistic regression, it was not an independent predictor of insulin-dependent gestational diabetes mellitus. 23 But in another study, it has been shown that grand multiparity was associated with an increased risk of diabetes.[24]

Some studies have shown that the fetuses whose mothers have experienced gestational diabetes mellitus are at increased risk for macrosomia and being large for gestational age which put them in the risk of birth trauma, shoulder dystocia, neonatal hypoglycemia and neonatal death.[25][26][27][28][29][30] We wondered if women with previous history of fetal or neonatal death are at increased risk of GDM. Although some results showed that history of prior neonatal death is a risk factor for GDM,[31] we did not find any association between fetal or neonatal death and GDM, as the difference between the mean of previous fetal or neonatal deaths in test and control groups did not reach the significant threshold (p=0.25 and p=0.20 respectively).

A study by Khooshideh et al. has reported that recurrent miscarriage is a risk factor for GDM.[5] In 1991, Hughes and colleagues conducted a study on 88 women with 3 or more miscarriages and stated that GDM and chronic hypertension is more frequent comparing with the control group.[32] In our study however, we could not find any significant relationship between abortion number and GDM (p=0.66). An explanation for this result could be that those studies were limited to just recurrent or more than three miscarriages but we considered all kinds of abortions in our study.

Several researchers reported that a woman’s own birth weight is inversely associated with her risk of gestational diabetes.[33][34][35] However, others proposed the need for further work to confirm these results.[36] The fetuses of women affected with GDM are at increased risk of preterm birth.[31][37][38] In this study however, we assessed whether women having previous history of preterm birth pregnancy are at increased risk for GDM. Although in our study the association of preterm birth and GDM was of borderline significance (p=0.055), these women may be at risk for GDM in later life.

Women with type 1 diabetes are at increased risk for thyroid diseases,[39] but it is not clear whether gestational diabetes patients are also susceptible. Amati et al. studied the effect of thyroid hormone status on insulin sensitivity and physical fitness after weight loss and exercise training and reported that these factors were improved in euthyroid but not in subclinical hypothyroid subjects.[40] In addition, it has been shown that subclinical hypothyroidism is associated with insulin resistance in patients with rheumatoid arthritis. [41] A study conducted by Velija-Asimi and associates on patients with subclinical hypothyroidism showed that treatment of this disease could decrease the levels of fasting insulin and fasting and postprandial glucose.[42] Also, the role of insulin resistance in gestational diabetes is reviewed by Mastrogiannis et al.[43] While insulin secretion is increased in normal pregnant women during pregnancy, gestational diabetes patients do not get this response.[44] Moreover, insulin sensitivity is decreased in these patients compared to healthy subjects.[45] All these evidences show that there may be a link between hypothyroidism and gestational diabetes.

In 2009, Torus et al. assesses the serum antioxidant levels in patients with subclinical and overt hypothyroidism and found that malondialdehyde, a lipid peroxidation marker, is increased in these patients compared with healthy subjects.[39] Similar results were reported elsewhere.[46] Chen and Scholl reviewed the role of oxidative stress in pregnancy and gestational diabetes mellitus.[47] Evidences showed that as oxidative stress increases and antioxidation defenses decrease, the secretion and action of insulin impair.[48] So it seems that the increase in oxidative stress or decrease in anti-oxidative system in hypothyroidism may interfere with insulin function.

There are evidences that physical activity could decrease the risk of gestational diabetes.[49] Besides, it has been shown that patients suffering from hypothyroidism are more prone to depression,[50] due to changes in catecholamine system of hypothalamus– pituitary–thyroid axis and brain serotonergic pathways, [51] which could limit their physical activity and increase the risk of gestational diabetes.

In a recent study by Liang et al., female mice were fed with high saturated fat diet one month before conception and during gestation to develop a mouse model of gestational diabetes. The test group developed more insulin resistance, lipid peroxidation and oxidation damage, features of gestational diabetes model than controls.[52] Comparing with non diabetics, women with gestational diabetes had higher serum level of triglyceride, as was reported by Enquobahrie and colleagues that each 20 mg/dl increase in TG was associated with a 10% increase in GDM risk.[53] Another study showed that LDL and triglyceride levels increased in gestational diabetes women.[54] Also, the levels of low density lipoproteins (LDL) in patients with gestational diabetes were evaluated by Rizzo et al. In comparison with the control group, women with gestational diabetes had higher LDL levels.55 Some studies have demonstrated that total cholesterol, LDL and triglycerides are increased in patients with hypothyroidism and treating these patients can decrease their levels.[56][57] The reason is that in hypothyroidism, the LDL receptors decrease and LDL accumulates in the serum.[58] Besides, thyroid hormones enhance the activity of lipoprotein lipase, which lowers the levels of triglycerides.[59] So in hypothyroidism, the triglycerides increase in serum and consequently, it can be a risk factor for developing gestational diabetes.

It has been shown that hypothyroidism is a risk factor for obesity.[60][61] In addition, lots of researchers reported that the risk of gestational diabetes increases as the BMI rise.[4][62] As a consequence, hypothyroid patients are at increased risk of getting gestational diabetes.

Our results showed that chronic hypertension is more prevalent in GDM women (p=0.002), which raises the possibility that hypertension is a predictor of GDM. Other studies reported similar results.[6][63][64] Caruso et al. reported that insulin sensitivity of women with chronic hypertension and GDM was approximately twofold lower than those with GDM only and they concluded that blood pressure is a strong predictor of insulin resistance.[65] Another study has reported the same results.[64] Metabolic syndrome is characterized by presence of hypertension, insulin resistance, glucose intolerance, dyslipidemia and central obesity. [66] Besides, it is regarded that chronic insulin resistance is central component of GDM.[67] According to aforementioned data, we conclude that women with gestational diabetes have an insulin resistance background. Some features of this background, such as hypertension show themselves earlier which can predict the occurrence of other elements, such as GDM, in later life. In addition to the fact that gestational diabetes is associated with hypertensive disorders, it has shown that subclinical hypothyroidism could lead to hypertension, mainly high diastolic pressure.[68] So there is one possibility that the association of gestational diabetes with hypertension, at least in part, is due to the more prevalence of hypothyroidism in gestational diabetes women.

In this study, we showed that maternal age, the birth weight of previous neonate, hypothyroidism, chronic hypertension and probably previous history of having a preterm birth are associated with GDM. Results of such studies can be used to predict the chance of GDM in healthy women and adopting preventive strategies in at-risk population. Although some studies suggest that universal screening appears to be the most reliable method of diagnosing GDM,[19] in 2009, the American Diabetes Association stated that screening a low-risk group of pregnant women is probably not cost-effective.[69] Therefore, identifying new risk factors for GDM can increase the sensitivity for selection of those women benefits from oral glucose tolerance test. Surely, more studies are needed to confirm our results.

## References

[1] Ferrara A, Hedderson MM, Quesenberry CP, Selby JV (2002). Prevalence of gestational diabetes mellitus detected by the national diabetes data group or the carpenter and coustan plasma glucose thresholds. Diabetes Care.

[2] Serlin DC, Lash RW (2009). Diagnosis and management of gestational diabetes mellitus. Am Fam Physician.

[3] (2004). American Diabetes Association.Gestational diabetes mellitus. Diabetes Care.

[4] Hossein-Nezhad A, Maghbooli Z, Vassigh AR, Larijani B (2007). Prevalence of gestational diabetes mellitus and pregnancy outcomes in Iranian women. Taiwan J Obstet Gynecol.

[5] Khooshideh M, Shahriari A (2008). Comparison of universal and risk factor based screening strategies for gestational diabetes mellitus. Shiraz EMed J.

[6] Hadaegh F, Tohidi M, Harati H, Kheirandish M, Rahimi S (2005). Prevalence of gestational diabetes mellitus in southern Iran (Bandar Abbas City).. Endocr Pract.

[7] HAPO Study Cooperative Research Group, Metzger BE, Lowe LP, Dyer AR, Trimble ER, Chaovarindr U, Coustan DR, Hadden DR, McCance DR, Hod M, McIntyre HD, Oats JJ, Persson B, Rogers MS, Sacks DA (2008). Hyperglycemia and adverse pregnancy outcomes. N Engl J Med.

[8] Odar E, Wandabwa J, Kiondo P (2004). Maternal and fetal outcome of gestational diabetes mellitus in Mulago Hospital, Uganda. Afr Health Sci.

[9] Crowther CA, Hiller JE, Moss JR, McPhee AJ, Jeffries WS, Robinson JS (2005). Australian Carbohydrate Intolerance Study in Pregnant Women (ACHOIS) Trial Group. Effect of treatment of gestational diabetes mellitus on pregnancy outcomes. N Engl J Med.

[10] Kaaja R, Rönnemaa T (2008). Gestational diabetes: pathogenesis and consequences to mother and offspring. Rev Diabet Stud.

[11] Karcaaltincaba D, Kandemir O, Yalvac S, Guvendag-Guven S, Haberal A (2009). Prevalence of gestational diabetes mellitus and gestational impaired glucose tolerance in pregnant women evaluated by National Diabetes Data Group and Carpenter and Coustan criteria. Int J Gynaecol Obstet.

[12] Ross G (2006). Gestational diabetes. Aust Fam Physician.

[13] Ogonowski J, Miazgowski T, Kuczynska M, Krzyzanowska-Swiniarska B, Celewicz Z (2009). Pregravid body mass index as a predictor of gestational diabetes mellitus. Diabet Med.

[14] Solomon CG, Willett WC, Carey VJ, Rich-Edwards J, Hunter DJ, Colditz GA, Stampfer MJ, Speizer FE, Spiegelman D, Manson JE (1997). A prospective study of pregravid determinants of gestational diabetes mellitus. JAMA.

[15] Jiménez-Moleón JJ, Bueno-Cavanillas A, Luna-del-Castillo JD, Lardelli-Claret P, García-Martín M, Gálvez-Vargas R (2000). Predictive value of a screen for gestational diabetes mellitus: influence of associated risk factors. Acta Obstet Gynecol Scand.

[16] Ben-Haroush A, Yogev Y, Hod M (2004). Epidemiology of gestational diabetes mellitus and its association with Type 2 diabetes. Diabet Med.

[17] Berg M, Adlerberth A, Sultan B, Wennergren M, Wallin G (2007). Early random capillary glucose level screening and multidisciplinary antenatal teamwork to improve outcome in gestational diabetes mellitus. Acta Obstet Gynecol Scand.

[18] Kieffer EC, Sinco B, Kim C (2006). Health behaviors among women of reproductive age with and without a history of gestational diabetes mellitus. Diabetes Care.

[19] Shamsuddin K, Mahdy ZA, Siti Rafiaah I, Jamil MA, Rahimah MD (2001). Risk factor screening for abnormal glucose tolerance in pregnancy. Int J Gynaecol Obstet.

[20] Khatun N, Latif SA, Uddin MM (2009). Risk factors for the development of gestational diabetes mellitus. Mymensingh Med J.

[21] Kim C, Sinco B, Kieffer EA (2007). Racial and ethnic variation in access to health care, provision of health care services, and ratings of health among women with histories of gestational diabetes mellitus. Diabetes Care.

[22] Roman H, Robillard PY, Hulsey TC, Laffitte A, Kouteich K, Marpeau L, Barau G (2007). Obstetrical and neonatal outcomes in obese women. West Indian Med J.

[23] Roman H, Robillard PY, Verspyck E, Hulsey TC, Marpeau L, Barau G (2004). Obstetric and neonatal outcomes in grand multiparity. Obstet Gynecol.

[24] Simmons D, Shaw J, McKenzie A, Eaton S, Cameron AJ, Zimmet P (2006). Is grand multiparity associated with an increased risk of dysglycaemia?. Diabetologia.

[25] Solomon CG, Willett WC, Carey VJ, Rich-Edwards J, Hunter DJ, Colditz GA, Stampfer MJ, Speizer FE, Spiegelman D, Manson JE (1997). A prospective study of pregravid determinants of gestational diabetes mellitus. JAMA.

[26] Weeks JW, Major CA, de Veciana M, Morgan MA (1994). Gestational diabetes: does the presence of risk factors influence perinatal outcome?. Am J Obstet Gynecol.

[27] Magee MS, Walden CE, Benedetti TJ, Knopp RH (1993). Influence of diagnostic criteria on the incidence of gestational diabetes and perinatal morbidity. JAMA.

[28] Casey BM, Lucas MJ, McIntire DD, Leveno KJ (1997). Pregnancy outcomes in women with gestational diabetes compared with the general obstetric population. Obstet Gynecol.

[29] Galerneau F, Inzucchi SE (2004). Diabetes mellitus in pregnancy. Obstet Gynecol Clin North Am.

[30] Schneiderman EH (2010). Gestational diabetes: an overview of a growing health concern for women. J Infus Nurs.

[31] Xiong X, Saunders LD, Wang FL, Demianczuk NN (2001). Gestational diabetes mellitus: prevalence, risk factors, maternal and infant outcomes. Int J Gynaecol Obstet.

[32] Hughes N, Hamilton E, Tulandi T (1991). Obstetric outcome in women after multiple spontaneous abortions. J Reprod Med.

[33] Innes KE, Byers TE, Marshall JA, Baron A, Orleans M, Hamman RF (2002). Association of a woman's own birth weight with subsequent risk for gestational diabetes. JAMA.

[34] Pettitt DJ, Jovanovic L (2007). Low birth weight as a risk factor for gestational diabetes, diabetes, and impaired glucose tolerance during pregnancy. Diabetes Care.

[35] Claesson R, Aberg A, Marsál K (2007). Abnormal fetal growth is associated with gestational diabetes mellitus later in life: population-based register study. Acta Obstet Gynecol Scand.

[36] Plante LA (2004). Low birth weight may not predict diabetes in pregnancy. Diabetes Care.

[37] Yogev Y, Langer O (2007). Spontaneous preterm delivery and gestational diabetes: the impact of glycemic control. Arch Gynecol Obstet.

[38] Lao TT, Ho LF (2003). Does maternal glucose intolerance affect the length of gestation in singleton pregnancies?. J Soc Gynecol Investig.

[39] Torun AN, Kulaksizoglu S, Kulaksizoglu M, Pamuk BO, Isbilen E, Tutuncu NB (2009). Serum total antioxidant status and lipid peroxidation marker malondialdehyde levels in overt and subclinical hypothyroidism. Clin Endocrinol (Oxf).

[40] Amati F, Dube JJ, Stefanovic-Racic M, Toledo FG, Goodpaster BH (2009). Improvements in insulin sensitivity are blunted by subclinical hypothyroidism. Med Sci Sports Exerc.

[41] Dessein PH, Joffe BI, Stanwix AE (2004). Subclinical hypothyroidism is associated with insulin resistance in rheumatoid arthritis. Thyroid.

[42] Velija-Asimi Z, Karamehic J (2007). The effects of treatment of subclinical hypothyroidism on metabolic control and hyperinsulinemia. Med Arh.

[43] Mastrogiannis DS, Spiliopoulos M, Mulla W, Homko CJ (2009). Insulin resistance: the possible link between gestational diabetes mellitus and hypertensive disorders of pregnancy. Curr Diab Rep.

[44] Persson B, Edwall L, Hanson U, Nord E, Westgren M (1997). Insulin sensitivity and insulin response in women with gestational diabetes mellitus. Horm Metab Res.

[45] Catalano PM, Huston L, Amini SB, Kalhan SC (1999). Longitudinal changes in glucose metabolism during pregnancy in obese women with normal glucose tolerance and gestational diabetes mellitus. Am J Obstet Gynecol.

[46] Nanda N, Bobby Z, Hamide A (2008). Oxidative stress and protein glycation in primary hypothyroidism. Male/female difference. Clin Exp Med.

[47] Chen X, Scholl TO (2005). Oxidative stress: changes in pregnancy and with gestational diabetes mellitus. Curr Diab Rep.

[48] Evans JL, Goldfine ID, Maddux BA, Grodsky GM (2003). Are oxidative stressactivated signaling pathways mediators of insulin resistance and betacell dysfunction?. Diabetes.

[49] Dode MA, dos Santos IS (2009). Non classical risk factors for gestational diabetes mellitus: a systematic review of the literature. Cad Saude Publica.

[50] Guimaraes JM, de Souza Lopes C, Baima J, Sichieri R (2009). Depression symptoms and hypothyroidism in a population-based study of middleaged Brazilian women. J Affect Disord.

[51] Danilo Q, Gloger S, Valdivieso S, Ivelic J, Fardella C (2004). Mood disorders, psychopharmacology and thyroid hormones. Rev Med Chil.

[52] Liang C, Decourcy K, Prater MR (2010). High-saturated-fat diet induces gestational diabetes and placental vasculopathy in C57BL/6 mice. Metabolism.

[53] Enquobahrie DA, Williams MA, Qiu C, Luthy DA (2005). Early pregnancy lipid concentrations and the risk of gestational diabetes mellitus. Diabetes Res Clin Pract.

[54] Sánchez-Vera I, Bonet B, Viana M, Quintanar A, Martín MD, Blanco P, Donnay S, Albi M (2007). Changes in plasma lipids and increased lowdensity lipoprotein susceptibility to oxidation in pregnancies complicated by gestational diabetes: consequences of obesity. Metabolism.

[55] Rizzo M, Berneis K, Altinova AE, Toruner FB, Akturk M, Ayvaz G, Rini GB, Spinas GA, Arslan M (2008). Atherogenic lipoprotein phenotype and LDL size and subclasses in women with gestational diabetes. Diabet Med.

[56] Mikhail GS, Alshammari SM, Alenezi MY, Mansour M, Khalil NA (2008). Increased atherogenic low-density lipoprotein cholesterol in untreated subclinical hypothyroidism. Endocr Pract.

[57] Pearce EN, Wilson PW, Yang Q, Vasan RS, Braverman LE (2008). Thyroid function and lipid subparticle sizes in patients with short-term hypothyroidism and a population-based cohort. J Clin Endocrinol Metab.

[58] Pearce EN (2004). Hypothyroidism and dyslipidemia: modern concepts and approaches. Curr Cardiol Rep.

[59] Lam KS, Chan MK, Yeung RT (1986). High-density lipoprotein cholesterol, hepatic lipase and lipoprotein lipase activities in thyroid dysfunction-- effects of treatment. Q J Med.

[60] Aronne LJ (2002). Classification of obesity and assessment of obesity-related health risks. Obes Res.

[61] Weaver JU (2008). Classical endocrine diseases causing obesity. Front Horm Res.

[62] Kwak SH, Kim HS, Choi SH, Lim S, Cho YM, Park KS, Jang HC, Kim MY, Cho NH, Metzger BE (2008). Subsequent pregnancy after gestational diabetes mellitus: frequency and risk factors for recurrence in Korean women. Diabetes Care.

[63] Roberts R (1998). Hypertension in women with gestational diabetes. Diabetes Care.

[64] Zetterstrom K, Lindeberg SN, Haglund B, Hanson U (2005). Maternal complications in women with chronic hypertension: a population-based cohort study. Acta Obstet Gynecol Scand.

[65] Caruso A, Ferrazzani S, De Carolis S, Lucchese A, Lanzone A, Paradisi G (1999). Carbohydrate metabolism in gestational diabetes: effect of chronic hypertension. Obstet Gynecol.

[66] Alberti KG, Eckel RH, Grundy SM, Zimmet PZ, Cleeman JI, Donato KA, Fruchart JC, James WP, Loria CM, Smith SC Jr (2009). International Diabetes Federation Task Force on Epidemiology and Prevention; Hational Heart, Lung, and Blood Institute; American Heart Association; World Heart Federation; International Atherosclerosis Society; International Association for the Study of Obesity. Harmonizing the metabolic syndrome: a joint interim statement of the International Diabetes Federation Task Force on Epidemiology and Prevention; National Heart, Lung, and Blood Institute; American Heart Association; World Heart Federation; International Atherosclerosis Society; and International Association for the Study of Obesity. Circulation.

[67] Lain KY, Catalano PM (2007). Metabolic changes in pregnancy. Clin Obstet Gynecol.

[68] Luboshitzky R, Aviv A, Herer P, Lavie L (2002). Risk factors for cardiovascular disease in women with subclinical hypothyroidism. Thyroid.

[69] American Diabetes Association (ADA). (2009). American Diabetes Association (ADA). Diagnosis and classification of diabetes mellitus. Diabetes Care.

